# MSC Manufacturing for Academic Clinical Trials: From a Clinical-Grade to a Full GMP-Compliant Process

**DOI:** 10.3390/cells10061320

**Published:** 2021-05-26

**Authors:** Chantal Lechanteur, Alexandra Briquet, Virginie Bettonville, Etienne Baudoux, Yves Beguin

**Affiliations:** 1 Laboratory of Cell and Gene Therapy, Department of Hematology, CHU of Liège, 4000 Liège, Belgium; alexandra.briquet@chuliege.be (A.B.); v.bettonville@chuliege.be (V.B.); e.baudoux@chuliege.be (E.B.); yves.beguin@chuliege.be (Y.B.); 2 Division of Hematology, Department of Medicine, CHU of Liège, University of Liège, 4000 Liège, Belgium

**Keywords:** cell therapy, MSC, GMP, ATMP manufacturing, mesenchymal stromal cells

## Abstract

Following European regulation 1394/2007, mesenchymal stromal cell (MSCs) have become an advanced therapy medicinal product (ATMP) that must be produced following the good manufacturing practice (GMP) standards. We describe the upgrade of our existing clinical-grade MSC manufacturing process to obtain GMP certification. Staff organization, premises/equipment qualification and monitoring, raw materials management, starting materials, technical manufacturing processes, quality controls, and the release, thawing and infusion were substantially reorganized. Numerous studies have been carried out to validate cultures and demonstrate the short-term stability of fresh or thawed products, as well their stability during long-term storage. Detailed results of media simulation tests, validation runs and early MSC batches are presented. We also report the validation of a new variant of the process aiming to prepare fresh MSCs for the treatment of specific lesions of Crohn’s disease by local injection. In conclusion, we have successfully ensured the adaptation of our clinical-grade MSC production process to the GMP requirements. The GMP manufacturing of MSC products is feasible in the academic setting for a limited number of batches with a significant cost increase, but moving to large-scale production necessary for phase III trials would require the involvement of industrial partners.

## 1. Introduction

Cell-based therapy is a fast-growing field including various cell products and indications. The first success for cell-based therapies was bone marrow (BM) transplantation around 60 years ago.

Since then, a better understanding of the immune system has allowed the development of diverse autologous and allogeneic cell therapies. Among these, mesenchymal stem or stromal cells (MSCs) are one of the most studied, with more than 1500 ongoing clinical trials (https://clinicaltrials.gov, accessed on 26 May 2021) investigating their regenerative properties and immunomodulatory potential. MSCs are evaluated in numerous indications, such as graft-versus-host disease (GVHD), autoimmune and inflammatory diseases (Crohn’s disease, etc.), heart failure, solid organ transplantation, liver or kidney failure, and bone diseases [1,2,3,4,5,6,7,8]. Most of them are phase I–II studies with only around 60 phase III clinical trials. While industrial partners are involved in these phase III studies (Caristem/Medipost/Seoul/South Korea, Prochymal/Osiris/Genzyme/MD/USA), most phase I–II trials are conducted by academic centers.

The Laboratory of Cell and Gene Therapy (LTCG) is a small structure within the department of clinical hematology at the University Hospital of Liège in Belgium. The hematology clinical unit has much experience with stem cell transplantation (bone marrow, peripheral blood stem cells, cord blood). Indeed, the first bone marrow transplantation was performed in 1982 (1997 for cord blood transplantation), and transplant numbers increased rapidly, reaching 100 in 1990, 500 ten years later, and more than 2000 by now. A cord blood bank was created in 1994 in the same department and around 3700 cord bloods have been processed and stored since then, with 169 procured for transplantation around the world. Due to the diversification of these activities, the LTCG cell facility was created in 2002 in order to ensure ongoing transplantation activities and allow for further technical development, such as MSC culture development, upscaling and later clinical-grade production.

Indeed, as already reported, we started in late 2006 to produce third-party mesenchymal stem cells (MSC) based on the clinical-grade expansion of MSC from BM samples obtained from healthy volunteer donors [9]. Cells were produced according to the European Group for Blood and Marrow Transplantation (EBMT) consortium recommendations for defining common procedures for MSC isolation and expansion, as well as common release criteria, facilitating multicenter trials with comparable MSC products. This clinical-grade process allowed us to initiate and run seven clinical trials with MSC intravenous (IV) infusion in different settings, including HSC transplantation (HCT) with myeloablative or non-myeloablative conditioning, cord blood transplantation (CBT), solid organ transplantation, and severe or refractory autoimmune disorders such as Crohn’s disease [10,11,12,13,14,15].

In the meantime, a new classification has come into place with European regulation 1394/2007. MSC and all ex vivo manipulated products (except for minimally manipulated products) were moved to the ATMP (advanced therapeutic medicinal products) category, and must be produced according to the good manufacturing practice (GMP) standards used for classical drugs. Recently, a new directive has been published including adaptations to the GMP standards of cell therapy products: “Guidelines on Good Manufacturing Practice specific to Advanced Therapy Medicinal Products” [16].

The present paper describes the mandatory global upgrade implemented to our MSC manufacturing process to make it compliant to GMP standards. The upgrade of facilities (classification, cleaning, environmental monitoring), staff evolution (dedicated tasks, training, clothing), process modifications (reagents, media-simulations, validation), quality controls and, of course, cost impacts related to the MSC activity will be discussed. Moreover, we present the validation results for the GMP transition of our existing MSC manufacturing process (frozen aliquots), as well as the validation of a new variant aiming to prepare low doses (30 × 10^6^) of fresh MSC for patients suffering from specific lesions (intestinal/colonic stricture, unhealed ulcer, perianal fistula) due to Crohn’s disease and treated by local injection. Lastly, GMP batch production will also be described and compared to previously produced clinical-grade batches.

## 2. Materials and Methods

### 2.1. Staff

GMP standards require clearly defined roles and responsibilities be outlined for the key personnel. This means that the responsibilities of production and quality control cannot be shared by the same person. A qualified person (QP) is also mandatory. The QP is an industrial pharmacist or equivalent who will have the responsibility of releasing the cell therapy products for clinical use. Additionally, a person in charge of quality assurance can be necessary depending on the size of the structure. If not, quality assurance tasks can be assumed or shared by the production manager, the QC manager or the qualified person. At the technical level, the same distinction between lab technicians involved in production or quality control tasks must exist.

In our previous structure, production and QC supervision were performed by the same manager and three lab technicians also shared production and QC tasks. As such, to be compliant to GMP standards, we had to hire a production manager, a part-time qualified person (pharmacist), a quality assurance manager, and three more lab technicians (one for instrumentation while working in aseptic conditions under laminar flow). Thus, from a team of four, we had to increase to nine people to ensure MSC activity according to GMP standards.

All personnel must also receive training on the principles of GMP that affect them, and receive initial and periodic training relevant to their tasks. As an example, even if the production staff were used to working in class A in C environments for clinical-grade production, manipulations in a class A in B environment are very different. The member of staff’s hands must stay under the laminar flow and instrumentation must be performed by a second operator or a logistician. Validation is performed during the process simulation (media fill) after the development of each new process (3 process simulations for validation of each new process) and repeated periodically (twice a year for each process and once a year for each operator).

The previously used clothing has also been completely replaced with a complete sterile suit with googles and boots with the body now completely covered. The gowning of personnel working in grade A/B areas was subjected to validation with microbial monitoring. Compliance with gowning requirements should be reassessed at least annually.

### 2.2. Premises and Equipment

According to GMP for ATMP regulation, premises must be qualified and adequately controlled to ensure an aseptic environment. The classification of clean rooms should be done according to the ISO 14644-1 rule.

The manufacturing of ATMP must be performed in a class A (laminar flow) in class B area. These areas are monitored for viable (air sampling, sedimentation and contact plates) and non-viable (particle count) contamination, air pressure, temperature and humidity.

Previously, our clinical-grade process of MSC production took place in a class A (laminar flow) in C area. Particle detection (non-viable monitoring) was checked twice a year, while viable monitoring was analyzed monthly (Table 1). Now, class A and B areas are monitored during all operation steps and also at rest. The frequency of environmental monitoring at rest depends on the class of the area. Viable and non-viable monitoring are performed weekly in class A and bimonthly in class B areas. Personnel working in A/B areas are monitored after critical operations and when leaving the A/B area. If microorganisms are detected in a grade A area, they should be identified to the species level, and their impact on product quality and the suitability of the premises for the intended operations should be assessed.

Al this necessitated new equipment (counters, air samplers), the use of many more contact plates, and many working hours, not only for sampling but also to analyze the results.

The cleaning of these areas is also a critical point. All the disinfection, cleaning products and materials must be sterile, and the process of cleaning/disinfection has to be thoroughly validated to demonstrate that the applied cleaning procedure effectively and reproducibly removes contaminants, residues from previous products and cleaning agents below a pre-defined threshold. Sterile products and materials are not mandatory for a class C area cleaning procedure, and full validation of the cleaning process is not necessary to maintain a class C classification of the room.

Equipment must be subjected to several qualification procedures: installation qualification (IQ), operational qualification (OQ) and performance qualification (PQ), in accordance with GMP requirements. Each of them must be extensively documented. Maintenance and reparation must also be documented. Before the acquisition of any new equipment, specifications must be defined and written as a URS (user requirement specification).

### 2.3. Raw Materials

On the contrary, the management of raw materials had to be considerably adapted to adhere to GMP requirements. Previously, reagents and disposables were checked for adequacy to their use. These verifications included correlation with the order form, expiration date, and sterility assessment when applicable, and the first-in first-out (FIFO) principle was applied for their use.

Now, each raw material is described in a specification (such as those in pharmacopoeia monographs for marketing/clinical trial authorization) including the quality requirements to ensure suitability for its intended use, as well as the acceptance criteria. The first step was thus to write specifications for all of the raw materials and to classify them into five categories according to their critical role in the process. The first category includes the most critical raw materials that are of animal origin, used in the class B environment and in close contact to the ATMP product (typically fetal bovine serum or FBS). Upon reception, raw materials are stored in quarantine until release according to their specifications by the QC or QA manager. Of course, the specifications of the critical raw materials (1st category) are more stringent than for the 5th category materials. In our case, this involved the writing of more than 200 specifications and the creation of a physical quarantine for all these materials. Besides this, SOPs and audits have also been set up for providers.

While enforcing GMP standards on our MSC banking activity, we introduced some changes in reagents and disposables that were not mandatory according to the regulation, but that we had found appropriate. First, we decided to move from T175 cm^2^ flasks to cellstacks (1, 2 or 5 levels) to limit the number of container manipulations (in incubators, under laminar flow) that increase the risk of contamination.

Second, antibiotic supplementation was also discontinued. Indeed, in our previous clinical-grade process, cells were cultured in 1% penicillin/streptomycin and, even if the residual P/S levels were insignificant due to thorough washing steps, the theoretical risk of allergic reactions persisted.

Finally, in accordance with the Note for Guidance on Minimizing the Risk of Transmitting Animal Spongiform Encephalopathy (TSE) Agents via Human and Veterinary Medicinal Products, the trypsin of porcine origin previously used to detach cells was replaced by a recombinant equivalent (TrypLE, Life Technologies, Bleiswijk, The Netherlands). However, gamma-irradiated FBS is still used as the source of growth factors in our MSC manufacturing process. Gamma-irradiation is used as a viral inactivation step that prevents contamination with adventitious agents.

### 2.4. Starting Material: Bone Marrow Collection

Allogeneic donor recruitment and bone marrow (starting material) collection are performed as described previously [9]. Indeed, the starting materials must be in accordance with Directive 2004/23/EC. If found to be eligible, the donor has to sign an informed consent form, and the marrow collection is then scheduled within 30 days of the screening visit using standardized prescription forms.

Quality requirements were established for the release of bone marrow starting materials. As these are fresh products, their release is performed as a two-step process: first, a provisional release based on donor eligibility criteria (physician advice together with biology and serology analyses plus donor consent), and second, a final release based on results obtained for the starting material itself that must comply with all the quality criteria defined in the specifications (serology, sterility, cell number, etc.). However, the bone marrow sample can proceed along the process after provisional release.

After collection, bone marrow is directly processed by the hematopoietic cell bank staff as already described [9]. Briefly, bone marrow is subjected to a fully automated and closed ficoll isolation procedure. The mononuclear cell suspension is then provisionally released and transferred to the ATMP manufacturing staff to be processed in a class A in B area.

### 2.5. Manufacturing Process

From the release of the mononuclear cell suspension to the end of the process, all manipulations are performed by the ATMP production staff in a class A in B environment with thorough environmental monitoring.

**Figure 1:**
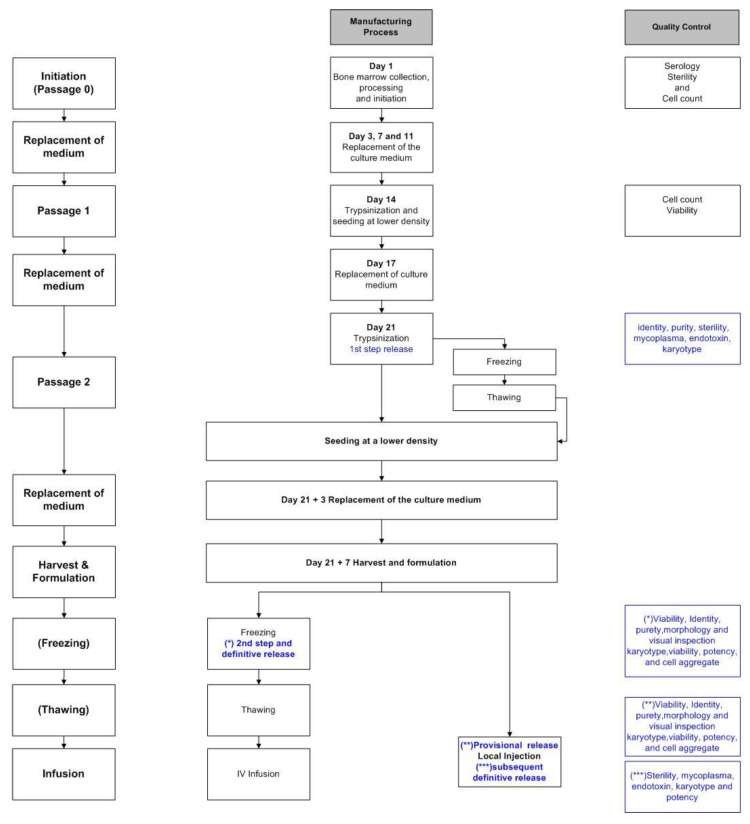
Manufacturing process and quality controls: from +/− 50 mL of initial fresh BM; MNC cells were isolated by automated Ficoll isolation and seeded in flasks or cellstacks. After passage 0 (P0) expansion during 14 days, cells were harvested and re-loaded in new cellstacks for P1 expansion. One week later, cells were harvested and re-loaded according to the same scheme for P2. Quality controls were performed at different stages of the process. After a first release at passage 2, cells can be either replated or immediately frozen (holding time). Fresh cells are harvested seven days later while frozen cells can be thawed and re-seeded in culture for seven more days before harvesting, and either frozen or formulated and freshly infused into patients. Cells from the same batch can be divided in the different pathways of the process.

The cell suspension is seeded in sterile tissue culture flasks (T-175 or CellSTACK, Corning (Corning B.V., Wiesbaden, Germany)) and the cell culture steps are the same as previously described [9], except that the media (DMEM-LGGLX (Fisher-Bioblock, Invitrogen, Merelbeke, Belgium) and 10% FBS (FBS, Hyclone, Perbio Sciences, Smithfield, UT, USA)) are free of antibiotics, and TrypLE is used instead of trypsin of porcine origin. Briefly, adherent precursors are selected by removing non-adherent cells and expanded with the regular replacement of culture media for two weeks until passage 1. After dilution at the appropriate density, cells are replated in new Cellstacks. On day 21, the cells are again nearly confluent and subjected to a second passage, and then either immediately replated or frozen for subsequent thawing and culture (Figure 1).

Around day 28 (or 7–8 days after thawing for P2-frozen cells), the MSCs are ready for harvesting. After washing and trypsinization, cells are resuspended in the harvesting solution (95% saline (0.9%) (Baxter S.A., Lessines, Belgium) and 5% human albumin (20%) (Alburex, CSL Behring GmBh, Marburg, Germany)). Two options are available at this stage for P2-frozen/thawed cells. The first option involves the intra-lesional injection of low doses of fresh cells into a Crohn’s disease patient. The cells are resuspended in harvesting solution (75% saline (0.9%) and 25% human albumin (20%)) at the concentration of 3.75 × 10^6^ cells/mL and transferred to syringes to be injected into patients (4 syringes of 2 mL/lesion). In the second option, the cells are resuspended at a concentration of 2 × 10^6^ cells/mL in harvesting solution (95% saline (0.9%) and 5% human albumin (20%)) and mixed 1:1 (volume to volume) with the freezing solution (60% saline (0.9%), 20% human albumin (20%) and 20% DMSO). The cell suspension is finally frozen following a controlled temperature program and stored in gaseous nitrogen.

### 2.6. Quality Controls

Donor recruitment, bone marrow collection, MSC expansion culture, freezing and quality controls were carried out according to new GMP SOPs. Starting with bone marrow collection, during all steps of culture and cryopreservation, the samples and cell containers (T-flasks, freezing bags) are labeled according to ISBT standards, ensuring the traceability of the cellular product is sustained. After manufacturing, the labeling of investigational ATMP must comply with the requirements of Regulation (EU) No. 536/2014.

All components (equipment, starting cellular material, reagents, materials, personnel and methods) used in the manufacturing process and quality controls are recorded in new batch records in storage and monitoring software applications specially developed for GMP processes and monitoring. All such software was subjected to proper validation (access authorization, functionalities, robustness).

Many quality control methods have been modified according to GMP requirements. Sterility was previously assessed via a microbial culture of supernatant (aerobes, anaerobes and fungi with Bactalert^®^ (bioMerieux, Durham, CA, USA); Microbiology department of the Hospital), mycoplasm**a** screening by luminometry (Mycoalert^®^ (Lonza, Verviers, Belgium); Microbiology department of the Hospital) and semi-quantitative endotoxin detection via a limulus test (European pharmacopeia 2.6.14, Pharmacy department of the Hospital). The microbiological quality controls have now been subcontracted to a GMP-accredited QC laboratory (Eurofins, Milano, Italy) according to thoroughly validated European Pharmacopea methods: E.P. 2.6.27 for the sterility assessment of cell suspensions, E.P. 2.6.7 Nucleic Amplification Technique (NAT) for the detection of mycoplasmas, and EP 2.6.14 for quantitative endotoxin detection with kinetic chromogenic detection.

The cell count method has also been modified, even if not mandatory, in order to strengthen the reproducibility. Indeed, we previously counted cells via trypan blue exclusion in a Neubauer cell counting chamber, which is an operator-dependent method. We have now moved to an automated method with a Nucleocounter (Chemometec, Hsinchu, Taïwan), which has been validated for cGMP cell manufacturing and works with acridine orange to label total cells and dapi to label dead cells.

The population doubling level (PDL) was calculated according to the formula PDL = 3.322 (log Y − log I), where Y = number of cells harvested and I = number of cells plated at P1. Doubling time (DT) was calculated according to the formula DT = *t* × log (2)/log (number of cells harvested/number of cells plated), where *t* is the time in hours between passage 1 and cell harvest.

Moreover, a mandatory visual inspection has been added to the panel of quality controls for the release of MSC products (E.P. 2.9.20 method).

Purity, identity, karyotype and potency are still evaluated via the same technical methods as are used in clinical-grade production [9].

Sampling has also been considerably extended. Before GMP implementation, 2 vials of frozen cells were stored at day 0 (mononuclear cells), passage 2, and harvest. According to GMP, the samples must now be retained for analytical purposes (reference samples) and for identification purposes (retention sample of a fully packaged unit from a batch of finished product). A reference sample should contain enough cells to permit at least two full analytical QCs on the batch. We also retain additional QC samples to be able to repeat QC analyses if necessary. Indeed, as shown in Table 2, 2 reference and 1 or 2 QC samples are retained at passage 2 and at harvest, both at 2–8 °C (supernatant) and −150 °C (cells). As a retention sample, we now keep photographs of all the units produced just before freezing or administration (in case of syringes with fresh cells) as it is not feasible to keep a full unit of the finished product purely for identification purposes. The samples are stored in monitored refrigerators or nitrogen tanks and their location is registered in a software application.

### 2.7. Release of MSC Products

GMP requires the release of the ATMPs by a qualified person who has to check that production and quality controls of the batch have been performed in accordance with the relevant requirements.

In all cases (frozen and fresh cells), the first release step is performed at passage 2 based on the QC described in Table 3 (sterility, mycoplasma, endotoxin, identity, purity, karyotype and potency). The frozen/thawed product is released definitively in a second step, when all QC results listed in Table 3 are available. On the contrary, when cells are injected fresh after harvesting, it is necessary to perform a provisional release (2nd step) based on the QC results at passage 2 and the preliminary results at harvest, including identity, purity, morphology, viability and visual inspection; definitive release (3rd step) is permitted when sterility, mycoplasma, endotoxin, karyotype and potency results are known (Table 3).

### 2.8. Thawing of MSC Products

As soon as a frozen MSC batch has passed the final release, any bag of this batch can be thawed at the LTCG and infused into a patient included in a clinical trial. Previously, cells were thawed and diluted in PBS (1:0.75 MSC:PBS) (Miltenyi Biotec, Bergisch Gladbach, Germany) as described previously [9]. The same procedure is applied now, except that we use saline instead of PBS, and the dilution ratio is slightly different (1:0.5 MSC:NaCl). Cell count and viability are assessed by the Nucleocounter method. The cell product is then transferred in an appropriately labeled sterile transfer bag and transported to the hematology ward for infusion into the patient.

### 2.9. Media Simulations to Demonstrate Asepsis

GMP standards require that media fill or process simulation tests be performed to demonstrate that all steps of the ATMP manufacturing process adequately prevent contamination and ensure asepsis. Briefly, a special sterile microbiological nutrient growth medium (Media fill (TSB), VWR international, Leuven, Belgium) that supports bacterial growth is used instead of all reagents and cells. Media fill tests are performed in conditions following as closely as possible routine manufacturing operations. At the end of the process, the final containers readied for release are incubated for one week at 22.5 °C and another week at 32.5 °C (as per Pharmaceutical Inspection Convention—PI007-6, 1 January 2011). No evidence of turbidity should be observed; the microbial culture must be sterile and the media fill solution must be checked for persisting fertility by inoculation of the defined strains of bacteria, which must then all grow.

Media simulations must be successfully achieved initially, and then re-assessed each year and after any significant modification of the facilities, equipment and process. All operators must also be qualified, and have their ability to work in aseptic conditions assessed initially in three consecutive runs and then once a year for each process in which they are involved.

## 3. Results

### 3.1. Process Validation

#### 3.1.1. Fresh Culture

Three large-scale clinical MSC cultures were initiated for validation of the GMP manufacturing process. As described in the supplemental data (Table S1), all QC assessments of MSCs at the end of these three cultures attained the pre-defined qualification criteria.

#### 3.1.2. Holding Step (Freezing at the Second Passage)

To validate the freezing/thawing steps, cells frozen at passage 2 were thawed and seeded in culture for one more week before harvesting and QC analysis. As described in the supplemental data (Table S2), the three harvested populations were compliant to their specifications. Deviations emerged due to monitoring issues, but none impacted the quality of the final product. These three batches were thus validated under the GMP manufacturing process of fresh MSC.

#### 3.1.3. Short-Term Stability of MSC Products after Thawing

In order to validate the maximum interval between the thawing and administration of the product, three MSC bags from three different donors were thawed and QC assessments were performed at T0, T + 1 h, T + 2 h and T + 4 h. As shown in the supplemental data (Table S3), all results obtained with T0 and T + 1 h samples met the acceptance criteria. However, for one of the three batches, the cell proliferation rates did not meet acceptance criteria at T + 2 h and T + 4 h post-thawing. Evaluations of immunosuppressive properties were thus not performed on these samples. We therefore validated one hour as the maximum delay between the end of the MSCs thawing step and their administration to the patient.

#### 3.1.4. Short-Term Stability of Fresh MSC Products

Cells frozen at passage 2 (from three different donors) were thawed and seeded in culture for one more week before harvesting and formulation in syringes. Syringes were stored for 1, 2, 3 or 4 h at room temperature. Cells were then passed through an endoscope guide, and evaluated for recovery, viability, potency and proliferation. As shown in the supplemental data (Table S4), all specifications were met as late as 4 h after cell formulation in the syringe. We thus validated 4 h as the maximum delay between the end of the MSCs’ formulation step and their administration to the patient.

#### 3.1.5. Stability Program

A stability program has also been initiated to verify that the product remains within its specifications when kept under the relevant storage conditions. For this purpose, one bag containing frozen MSC is thawed and tested each year. Testing is performed one hour after thawing (maximum authorized time between thawing and infusion) and includes cell recovery, viability, sterility, mycoplasma, endotoxin, identity, purity, morphology, potency and ability to re-proliferate after one week in culture. At this point, we have been validating the stability of our MSC nitrogen storage for 3 years (Table 4).

Recovery represents the ratio of thawed MSCs to frozen MSCs. The proliferation rate is defined as the ratio between the number of cells obtained after one week of culture post-thawing and the number of thawed cells immediately recovered after thawing. Potency is calculated as the inhibition of activated PBMC proliferation obtained by co-culturing with MSCs, demonstrating their immunosuppressive properties in vitro.

#### 3.1.6. MSC Manufacturing

After all these set up and validation steps, the legal authorities granted us GMP certification and authorization for ATMP manufacturing according to our new GMP SOPs. Since then, 10 manufacturing runs have been initiated to produce MSCs (including the 3 validation runs), but 3 were aborted due to microbial contamination—1 in the collected BM and 2 at the first passage.

Different MSC production pathways have been validated for the final use of the cells, i.e., IV or the local injection of frozen or fresh cells, respectively (Figure 1). Moreover, cells from the same batch can be divided into the different pathways of the process (Table 5), with a lot of P2 aliquots still in process, awaiting freezing for subsequent culturing and harvesting. However, all harvested cells have been fully released, according to their compliance with specifications.

#### 3.1.7. Cost Increase with GMP Manufacturing

The implementation of GMP standards in the MSC production process generates substantial additional costs. Comparative evaluations of the costs involved in the production of one GMP-grade bag of MSCs show that they are 2.5 times higher than those for a clinical-grade bag (Table 6). Moreover, costs are further increased for MSCs injected fresh in small quantities for the local treatment of Crohn’s disease lesions. Indeed, the cells are then subjected to a holding step at P2 before being thawed in small quantities, and then cultured for one more week before harvest and formulation.

While costs of reagents and disposables (gowning, cellstacks, cleaning reagents) are moderately increased, most of the cost increase is due to staff working time and externalized quality controls. Indeed, we had to hire a full-time production manager, a part-time qualified person, a quality assurance manager, and three more lab technicians in order to separate production and QC tasks, and to perform additional tasks such as working under the laminar flow, gowning, the cleaning of facilities after each manipulation, thorough environmental monitoring during operations, etc.

In addition, quality assurance tasks have dramatically increased due to the adaptation of all existing SOPs and the writing of many new SOPS and specifications for all reagents and disposables. Quality assurance tasks also include the description and follow-up of all deviations, out of specifications and change controls. The tasks dedicated to equipment management have also substantially increased in magnitude.

The increased QC costs are mainly due to the externalization of sterility, mycoplasma and endotoxin analysis to a GMP-accredited lab, and to the intensive environmental monitoring of disposables, the performance of tests, and time required to analyze QC data. Additional costs also include media fill tests (process and operator qualification) and the validation of externalized QC control methods.

## 4. Discussion

To supply MSCs for the rapidly increasing number of clinical trials progressively moving to phase III, there is an urgent need for standardized methods to produce, control and release MSCs within large-scale manufacturing processes. The manufacturing of MSCs and other ATMPs requires compliance with GMP standards, and in particular with new guidelines on good manufacturing practice specific to advanced therapy medicinal products [16].

In this paper, we report on our efforts to implement GMP standards in our allogeneic MSC manufacturing process [9], which allowed us to obtain a GMP license in an academic setting. Specific adaptations were made to staff organization, the quality control and release of the cellular products, and the monitoring of the premises and equipment. The validation and stability of the upgraded GMP process are also described. The results of the first 10 GMP-grade MSC batches produced in our facility are also discussed. In addition, we provide an estimate of the increase in costs generated by the GMP upgrade of our MSC manufacturing process.

Very few changes were implemented in the technical process, and in particular in the raw materials used. Antibiotics and trypsin (of porcine origin) were omitted, but the use of gamma-irradiated FBS was maintained despite the increasing number of serum-free alternatives available on the market (platelet lysate, xeno-free media, chemically defined media). It should be noted that the FDA recently indicated that over 80% of the 66 investigational new drug applications for MSC products described the use of FBS during manufacturing [17]. For availability and safety reasons, serum-free media, such as human platelet lysate or xeno-free media, should be used in the manufacture of human ATMP, but the impacts of these on the properties of cultured MSCs has not yet been established.

Human platelet lysate (HPL) is an acceptable and frequently used alternative to serum in MSC culture media. HPL is known to improve MSC recovery by increasing cell proliferation compared with serum. This can be an advantage if we consider the costs of production, as fewer batches will be necessary to obtain better cell yields. However, we must keep in mind that the excessive proliferation of MSCs is not advisable, knowing that MSC potency can be lost after a certain number of population doublings, with the development of cell senescence. Thus, limiting the number of population doublings to less than 20 seems appropriate [18,19,20]. Moreover, it is not really clear by now whether MSCs grown in an HPL environment have the same biological properties as MSCs obtained in serum-containing media. This is particularly the case for immunomodulatory properties, with some studies reporting their maintenance when replacing FBS with HPL, while others reported decreased immunosuppressive and differentiation capacities [17,21,22]. Microarray analyses disclosed the up- or downregulation of many genes involved in the differentiation, adhesion and migration of MSC [23]. An opinion article concerning safety issues and the standardization of HPL production has just been published by the ISBT Working Party that proposes recommendations for manufacturing and quality management in line with the regulations related to biological products and ATMPs [24].

Commercially available serum-free media (Miltenyi Biotec, StemCell Technologies, GE, among others) also demonstrate enhanced proliferation with the generation of a population of smaller human MSCs. As MSCs are known to be mainly trapped in the lung after IV infusion, smaller cells could better escape through the lung and migrate to injured sites [17], thereby also reducing the risk of pulmonary embolism [23]. However, there is some evidence that MSC trapping in the lungs is necessary for the activation of the macrophage cascade and the paracrine immunosuppressive effects of MSC [25]. Bakopoulou et al. also demonstrated that dental MSCs cultured in xeno-free media showed differences in morphology, downregulation of some markers (CD146, CD105, stro-1, etc.) and the upregulation of osteogenic markers, as compared to MSCs grown in FBS-containing media [26].

Since the ISCT position paper [27] defining the minimal criteria for the identification of MSCs (fibroblastic morphology, plastic adherence, differentiation capacity) with the description of a minimal panel of markers (>95 expression of CD105, CD73 and CD90 and lack of expression of hematopoietic markers), no consensus has been obtained on a specific MSC phenotype.

Potency assessment is another challenge frequently brought up by regulatory authorities. Indeed, a potency assay really mimicking the in vivo effects of MSCs is an unmet need [28]. Mixed lymphocyte reaction (MLR) and similar approaches (PBMC stimulation assays) are still the gold standard used by us and most groups studying MSCs [29]. However, these methods may not be robust enough to be used in a potency release assay. Bloom et al. [30] showed that substituting one PBMC donor for another completely changes the magnitude of the immunosuppressive effects induced by MSCs. Such variability stems from the degree of histocompatibility (mismatch) between responder cells (PBMCs or lymphocytes) and MSCs. Several variations in standard inhibition assays have been proposed [31,32,33] to increase robustness, such as pooling cells from several donors as responder cells, adding a titration curve (different MSC to responder cell ratios), use of a reference MSC product, use of a reference responder cell line (T cell line Karpas 299), or the MSC-stimulated induction of Treg cells. However, most studies comparing in vitro versus in vivo immunomodulation were not able to find satisfactory correlations [34,35,36,37]. Markers of MSC activation (CD200, TNF-αR, IDO, PD-L1) have also been proposed as markers of MSC immunosuppressive properties. In particular, IDO is known to be a critical factor for human MSC-mediated immunomodulation, and is not expressed in resting MSCs [37].

An interesting approach has been developed by Guan et al., who compared protein expression between resting and IFNγ-licensed MSCs, and identified the intracellular mediator IDO-1 and surface molecule PD-L1 as interesting candidate molecules for potency assessment [38]. Indeed, they showed that IDO and PDL-1 expression by IFNγ-licensed MSCs correlated with their suppression ability. This test only requires routine flow cytometry, does not need third-party T lymphocytes or PBMC, and has a duration time of a few hours, as opposed to 4–7 days for standard proliferation assays. Moreover, it takes into account both the paracrine (soluble mediator IDO-1) and cellular (cell contact with PD-L1) pathways of immunomodulation effects [38].

Due to the low number of batches (10) produced so far and the variability due to production via different pathways, it was not feasible to perform statistical analyses comparing clinical-grade and GMP-grade MSC batches. However, if we consider the five GMP batches that were manufactured freshly (without a holding step) up to the final harvest, and compare them to our historical group of clinical-grade MSCs [9], no substantial modifications of the results are apparent in terms of recovery (highly variable from donor to donor) and QC results and compliance. We did not increase overall cell yields by moving from clinical-grade cultures in T-flasks to GMP-grade cultures in cellstacks. Indeed, the manufacturing capacity remains limited by available space, number of incubators, laminar flows and staff. The PDLs were in the same range as those derived with T-flasks or cellstacks. As described in our previous publication [9], the mean PDL in T-flasks was 4.7 for both fresh cultures and cultures with a frozen holding step. As shown in Table 5, the PDLs were a bit higher in our new process (mean 5.45), but this was only seen for fresh cultures and a limited number of batches. The yields are highly variable from one culture to another, and clinical studies may also require different numbers of cells. Patients suffering from specific lesions due to Crohn’s disease are treated by local injection of 30 × 10^6^ cells/lesion, while patients treated intravenously for COVID19-related ARDS receive three injections of 3 × 10^6^ cells/kg 3 days apart, i.e., a total of approximately 800 × 10^6^ cells (for 85 kg BW), representing six MSC bags. Depending on the donor, our manufacturing process may yield 20 bags of 150 × 10^6^ cells each (batch two in Table 5), or as little as 3 bags (batch one in Table 5).

Upgrading our MSC manufacturing process to meet the GMP requirements induced substantial cost increases, mainly due to staff costs, QC externalization and extensive environmental monitoring. If we consider the simple process of producing one frozen bag of MSCs, the cost is multiplied by at least 2.5 when applying GMP rules. Additionally, this does not include one shot, or costs such as the validation of QC methods in external laboratories and staff qualifications.

Moreover, if we consider the additional processes involved in GMP-grade MSCs, particularly when small quantities of freshly injected cells are prepared for the local treatment of lesions in Crohn’s disease, costs are increased further. Furthermore, the costs would probably be much higher if we implemented the production of autologous instead of allogeneic MSCs, as one MSC batch would only treat one patient (and the culture yields would probably be much lower).

Harrison et al. recently published an interesting study on the economic feasibility of cell micro factories for the production of 2500 doses of 7.5 × 10^6^ cells per year, which is in the same order of magnitude as our capacity [39]. The production process involved three passages in monolayer expansion platforms. The use of serum-free (SFM) instead of serum-containing (SCM) media increased the growth kinetics, reduced the costs, and improved the consistency between batches. However, this expansion potential may be limited by the maximum numbers of population doublings that can maintain adequate MSC functions.

## 5. Conclusions

In conclusion, we report here the successful GMP upgrade of our allogeneic MSC manufacturing process, with a description of all aspects concerning staff, premises, reagents, processes, quality controls and costs. The results of the validation and stability studies and the production of the first 10 batches are also described. The GMP manufacturing of MSC products intended for systemic or local administration is feasible in the academic setting for a limited number of batches, but moving to the large-scale production necessary for phase III trials would probably require the involvement of industrial partners. Further improvements will be realized by moving to serum-free media, attaining a better understanding MSCs mechanisms of action, and improving potency assays.

## Figures and Tables

**Table 1:** Comparison between environmental monitoring performed in clinical-grade and in GMP-certified manufacturing processes. Frequency and specifications are described for viable and non-viable monitoring.

Class		A	B	C	D
**Non-viable monitoring (particles)**					
***Frequency***					
**Clinical process**		NA	NA	Half-yearly	
**GMP process**		In operation weekly	In operation bimonthly		
Specifications (*)					
**At rest**	≥0.5 µm	3520	3520	352,000	3,520,000
	≥5 µm	20	29	2900	29,000
**In operation**	≥0.5 µm	3520	352,000	3,520,000	NA
	≥5 µm	20	2900	29,000	NA
**Viable monitoring (bacterial CFU)**					
***Frequency***					
**Clinical process**		NA	NA	Monthly	
**GMP process**		In operation weekly	In operation bimonthly		
***Specifications***					
**Air sampling (CFU/m^3^)**		<1	10	100	200
**Sedimentation (CFU/4H)**		<1	5	50	100
**Contact plate (CFU/plate)**		<1	5	25	50

(*) maximum permitted airborne particles/m^3^.

**Table 2:** Type and number of samples retained at each step of the culture.

	Clinical Process	GMP Process	
		Reference	Quality Control
**Initiation**	2 at ≤ −150 °C	2 at ≤ −150°C	
**Passage 2**	2 at ≤ −150 °C	2 at 2–8 °C (supernatant)	2 at 2–8 °C (supernatant)
		2 at ≤ −150 °C (viable cells)	2 at ≤ −150 °C (viable cells)
**Harvest**	2 at ≤ −150 °C	4 at 2–8 °C (supernatant)	4 at 2–8 °C (supernatant)
		2 at ≤ 150 °C (viable cells)	2 at ≤ −150 °C (viable cells)

**Table 3:** QC, specifications and release of the cells.

Test	Method	Release Criteria			
		First Common Step	Final Release Frozen Product	Provisional Release Fresh Product	Final Release Fresh Product
**Sterility**	E.P. 2.6.27	Culture negative at the limit of detection	Culture negative at the limit of detection	NA	Culture negative at the limit of detection
**Mycoplasma**	E.P. 2.6.7	Absence	Absence	NA	Absence
**Endotoxin**	E. P. 2.6.14	<2.5 UI/mL	<2.5 UI/mL	NA	<2.5 UI/mL
**Identity**	Phenotype by FACS	CD90 > 80% CD105 > 80% CD73 > 80%	CD90 > 95% CD105 > 95% CD73 > 95%	CD90 > 95% CD105 > 95% CD73 > 95%	NA
**Purity**	Phenotype by FACS	CD14 < 2% CD34 < 2% CD45 < 2% CD3 < 1% Total < 2%	CD14 < 2% CD34 < 2% CD45 < 2% CD3 < 1% Total < 2%	CD14 < 2% CD34 < 2% CD45 < 2% CD3 < 1% Total < 2%	NA
**Karyotype**	Cell culture	Absence of clonal chromosomal structure and/or number abnormalities	Absence of clonal chromosomal structure and/or number abnormalities	NA	Absence of clonal chromosomal structure and/or number abnormalities
**Viability**	Nucleocounter	NA	≥80%	≥80%	NA
**Potency**	MLR by FACS	NA	>25% Inhibition of the proliferation of activated PBMCs	NA	>25% Inhibition of the proliferation of activated PBMCs
**Morphology**	Microscopic observation	NA	Fibroblastic	Fibroblastic	NA
**Cell aggregate**	Nucleocounter	NA	<25%	NA	NA
**Visual inspection**	European Pharmacopoeia 2.9.20	NA	Absence of visible particle	Absence of visible particle	NA
**Freezing temperature curve**	Control of the freezing temperature curve	NA	Conform to the programmed temperature curve	NA	NA

**Table 4:** Validation of QC analysis of MSCs thawed after three-year storage in nitrogen.

Test		Specification	Results	Conformity
**Recovery (%)**		60% 69%	69%	Compliant
**Viability (%)**		60% 76%	76%	Compliant
**Sterility**		Sterile	Sterile	Compliant
**Endotoxin**		<2.5 UI/mL	<0.15 UI/mL	Compliant
**Mycoplasma**		Absent	OK	Compliant
**Identity**	**CD90**	>95%	98.3%	Compliant
	**CD105**	>95%	99.5%	Compliant
	**CD73**	>95%	96.7%	Compliant
**Purity**	**CD14**	<2%	0%	Compliant
	**CD34**	<2%	0%	Compliant
	**CD45**	<2%	0.05%	Compliant
	**CD3**	<1%	0%	Compliant
	**Total**	<2%	0.05%	Compliant
**Morphology**		Fibroblastic	Fibroblastic	Compliant
**Proliferation rate**		>1	1.44	Compliant
**Potency**		>25%	63.5%	Compliant

**Table 5:** MSC manufacturing.

Batch Number	P0 (Cells × 10^6^)	P1 (Cells × 10^6^)	P2 (Cells × 10^6^)	Unfrozen Seeded Cells (×10^6^)	Harvested Cells (×10^6^)	PDL (P1-Harvest)	N-Frozen Aliquots at P2	Frozen Cells/Aliquot (×10^6^)	N Thawed Bags	Post-Thaw Cells (×10^6^)	Post-Thaw Harvested Cells (×10^6^)
**1**	167.0	21.2	113.4	63.8	331.0	4.81					
							1	49.7	1	30.8	103.8
**2**	1330.0	525.0	823.5	204.0	1500.0	4.89					
							4	106.0 168.0 168.0 168.0	3	102.0 159.0 122.0	449.0 411.0 416.0
**3**	209.6	51.0	335.5	102.0	970.0	5.91					
							2	112.5 112.5	2	92.5 103.8	795.0 867.0
**4**	323.0	154.8	932.5	204.0	1552.6	6.04					
							13	2 × 136.0 1 × 147.0 10 × 30.0	1 2	106.0 25.1; 27.7	618.0 123.0; 154.5
**5**	176.4	40.8	220.0				6	33.7	1	20.3	93.3
**6**	688.8	207.2	1727.0				21	3 × 190.0 6 × 45.0 2 × 190.0 10 × 40.0	1	19.6	100.2
**7**	402.6	86.7	580.0	204.0	1463.5	5.60					
							4	1 × 86.0 3 × 89.0			
**Mean**	444.0	155.2	674.6	155.5	1163.0	5.45					

Results are shown for the 7 MSC batches successfully manufactured according to the GMP-compliant process and their transformation via the different pathways of the process. PDLs are calculated only for fresh cultures (without a holding step) according to the following: PDL = 3.322 (log Y − log I), where Y = number of cells harvested and I = number of cells inoculated at P1.

**Table 6:** Comparative costs of clinical-grade and GMP-grade processes.

Manufacturing Costs (*)	**Clinical-Grade**	**GMP-Grade (Fold)**
**Staff (manufacturing and QC) (28%)**	1	3.6
**Reagents and disposables (49%)**	1	1.9
**Externalized QC (sterility, mycoplasma, endotoxin) (11%)**	1	5.3
**Internal QC (phenotype, karyotype, potency)**	1	1
**Global process (staff, manufacturing and QC)**	1 (2633 EUR/bag)	2.5 (6647 EUR/bag **)
Extra Costs (*)	**Clinical-Grade**	**GMP-Grade**
**Equipment**	1	1.5
**Environmental monitoring (routine only, not in operation)**	1	7.9
**QA staff**	1	2
**Externalized QC method validation**	0	EUR ~17,000
**Media simulations**	0	EUR ~4000/run (EUR 12,000/validation and EUR 8000/year

(*) Manufacturing costs considering a standard culture process in 28 days without a holding step and ending with the banking of frozen aliquots. The extra costs include the fixed costs involved in maintaining the propriety of the structure, as well as costs related to QC method validation and media simulations. (**) One bag of MSCs contains between 80 and 190 × 10^6^ cells.

## Data Availability

Data is contained within the article or Supplementary Material.

## Supplementary Materials

The following are available online at https://www.mdpi.com/article/10.3390/cells10061320/s1. Table S1: Validation of fresh cultures (without holding step), Table S2: Validation of holding step, Table S3: Short-term stability of MSC products after thawing and 1, 2 or 4 h thereafter, Table S4: Short-term stability of fresh MSC products after formulation and 1, 2, 3 and 4 h thereafter.

## Abbreviations

ATMP	Advanced therapeutic medicinal product
BM	Bone marrow
CBT	Cord blood transplantation
DMSO	Dimethyl sulfoxide
DT	Doubling time
EBMT	European Group for Blood and Marrow Transplantation
EP	European pharmacopeia
FBS	Fetal bovine serum
FDA	Food and drug administration
FIFO	First-in first-out
GMP	Good manufacturing practice
GVHD	Graft versus host disease
HCT	Hematopoietic cell transplantation
HPL	Human platelet lysate
HSA	Human serum albumin
IDO	Indolamine oxidase
IFNγ	Interferon gamma
ISBT	International society of blood transfusion
ISCT	International Society of Cellular Therapy
IQ	Installation qualification
IV	Intravenous
LTCG	Laboratory of Cell and Gene Therapy
MLR	Mixed Lymphocyte Reaction
MSC	Mesenchymal stem/stromal cells
NaCl	Sodium chloride
NAT	Nucleic amplification technique
OQ	Operational qualification
PBMC	Peripheral blood mononuclear cells
PDL	Population doubling level
PDL-1	Programmed death ligand 1
PQ	Performance qualification
QA	Quality assurance
QC	Quality control
QP	Qualified person
SCM	Serum-containing media
SFM	Serum-free media
SOP	Standard operating procedure
TSE	Transmission of spongiform encephalopathy
URS	User-specific specifications
