# Supplementary material for: MSC Manufacturing for Academic Clinical Trials: From a Clinical-Grade to a Full GMP-Compliant Process
Source: Cells. 2021 May 26;10(6):1320. doi: 10.3390/cells10061320 (PMC8227789; doi:10.3390/cells10061320)
Supplement: Supplementary file 1 [file cells-10-01320-s001.zip › cells-1220813-supplementary.pdf]

Supplemental data

Table S1. Validation of fresh cultures (without holding step). Results of 3 large-scale MSC cultures for GMP process validation. QC results are shown for each culture step. Potency is calculated as inhibition of activated PBMC proliferation in co-culture with MSC.

Validation run number			1	2	3	
	**Test**	**Specifications**	**Results**	**Results**	**Results**	**Conformity**
**P0**	Initial cell number	NA	167 x 10^6	1,330 x 10^6	210 x 10^6	NA
**P1**	Harvested cells	NA	21 x 10^6	525 x 10^6	51 x 10^6	NA
**P2**	Harvested cells	NA	113 x 10^6	824 x 10^6	336 x 10^6	NA
	DT (hours) P1-P2	NA	69.5	83.4	61.8	NA
	PDL P1-P2	NA	2.4	2.0	2.7	NA
	Sterility	Sterile	OK	OK	OK	Compliant
	Mycoplasma	Absence	OK	OK	OK	Compliant
	Endotoxins	< 2.5 UI / ml	OK	OK	OK	Compliant
	Identity (Phenotype) (%)	CD90 > 80%<br>CD105 > 80%<br>CD73 > 80%	92.0<br>96.0<br>93.7	98.4<br>98.0<br>97.7	98.7<br>97.7<br>97.8	Compliant
	Purity (Phenotype) (%)	CD14 < 2%<br>CD34 < 2%<br>CD45 < 2%<br>CD3 < 1%<br>Total < 2%	0<br>0.13<br>0<br>0<br>0.13	0<br>0<br>0<br>0<br>0	0<br>0<br>0<br>0<br>0	Compliant
	Karyotype	Normal	OK	OK	OK	Compliant
**Harvest**	Harvested cells	NA	331 x 10^6	1,500 x 10^6	970 x 10^6	NA
	DT (hours) P2-harvest	NA	70.7	58.4	43.8	NA
	PDL P2-harvest	NA	2.4	2.9	3.2	NA
	Sterility	Sterile	OK	OK	OK	Compliant
	Mycoplasma	Absence	OK	OK	OK	Compliant
	Endotoxins	< 2.5 UI / ml	OK	OK	OK	Compliant
	Identity (Phenotype) (%)	CD90 > 95%<br>CD105 > 95%<br>CD73 > 95%	96.5<br>99.8<br>99.3	99.3<br>99.3<br>99.6	99.6<br>99.7<br>99.3	Compliant
	Purity (Phenotype) (%)	CD14 < 2%<br>CD34 < 2%<br>CD45 < 2%<br>CD3 < 1%<br>Total < 2%	0<br>0.04<br>0.07<br>0<br>0.11	0.22<br>0<br>0<br>0<br>0.22	0<br>0.08<br>0<br>0<br>0.08	Compliant
	Karyotype	Normal	OK	OK	OK	Compliant
	Viability (%)	≥ 80%	95	94 / 95 (*)	95	Compliant
	Potency (%)	> 25%	59	55	48	Compliant
	Morphology	Fibroblastic	Fibroblastic	Fibroblastic	Fibroblastic	Compliant

DT: Doubling time; PDL: Population doubling level
(*) First / Second harvest

Table S2. Validation of holding step. Results of 3 large-scale MSC cultures for validation of holding step in GMP process. Potency is calculated as inhibition of activated PBMC proliferation in co-culture with MSC.

**Validation run number**			**1**	**2**	**3**	
	**Test**	**Specifications**	**Results**	**Results**	**Results**	**Conformity**
**P2**	Viability (%)	NA	63	78	88	NA
	Seeded cells (thawed)	NA	31×10^6	117×10^6	93×10^6	NA
**Harvest**	Harvested cells	NA	104×10^6	449×10^6	795×10^6	NA
	DT (hours)	NA	95.9	86.4	17.4	NA
	PDL	NA	1.8	1.9	3.1	NA
	Sterility	Sterile	OK	OK	OK	Compliant
	Mycoplasma	Absence	OK	OK	OK	Compliant
	Endotoxin	< 2.5 UI / ml	OK	OK	OK	Compliant
	Identity (Phenotype) (%)	CD90 > 95%	98.6	99.6	99.7	Compliant
		CD105 > 95%	99.2	99.5	99.8	
		CD73 > 95%	98.5	98.8	99.3	
	Purity (Phenotype) (%)	CD14 < 2%	0	0	0	Compliant
		CD34 < 2%	0.3	0	0.03	
		CD45 < 2%	0	0	0	
		CD3 < 1%	0	0	0	
		Total < 2%	0.3	0	0.03	
	Karyotype	Normal	OK	OK	OK	Compliant
	Viability (%)	≥ 80%	94	94	97	Compliant
	Potency (%)	> 25%	56	58	61	Compliant
	Morphology	Fibroblastic	Fibroblastic	Fibroblastic	Fibroblastic	Compliant

DT: Doubling time; PDL: Population doubling level

Table S3. Short-term stability of MSC products after thawing and 1, 2 or 4 hours thereafter. Results for short-term stability are shown for 3 different MSC bags immediately after thawing (T0) and 1 (T1), 2 (T2) or 4 (T4) hours later. Recovery represents the ratio of thawed MSC/frozen MSC at the different times. Proliferation is defined as the ratio of number of cells after one week of post-thawing culture / number of cells recovered immediately after thawing. Potency is calculated as inhibition of activated PBMC proliferation in co-culture with MSC.

**Validation run number**			**1**	**2**	**3**	**Validation**
	**Tests**	**Specification**	**Results**	**Results**	**Results**	**Conformity**
**At thawing**	Sterility T0	Sterile	OK	OK	OK	Compliant
	Mycoplasma T0	Absent	OK	OK	OK	Compliant
	Endotoxin T0	< 2.5 UI/ml	OK	OK	OK	Compliant
	Identity T0 (%)	CD90 > 95%	95.5	98.9	99.2	Compliant
		CD105 > 95%	99.2	99.0	98.2	Compliant
		CD73 > 95%	98.4	98.3	98.9	Compliant
	Purity T0 (%)	CD14 < 2%	0	0.05	0.35	Compliant
		CD34 < 2%	0.02	0.30	0.55	Compliant
		CD45 < 2%	0	0.30	0	Compliant
		CD3 < 1%	0	0	0	Compliant
		Total < 2%	0.02	0.64	0.90	Compliant
	Viability T0 (%)	≥ 60%	80	72	87	Compliant
	Viability T1 (%)	≥ 60%	83	70	88	Compliant
	Viability T2 (%)	≥ 60%	78	71	90	Compliant
	Viability T4 (%)	≥ 60%	77	71	85	Compliant
	Recovery of viable cells T0 (%)	≥ 60%	81	71	98	Compliant
	Recovery of viable cells T1 (%)	≥ 60%	88	83	90	Compliant
	Recovery of viable cells T2 (%)	≥ 60%	79	69	96	Compliant
	Recovery of viable cells T4 (%)	≥ 60%	73	83	100	Compliant
**After 8 days of culture**	Proliferation T0	> 1	1.84	2.02	4.83	Compliant
	Proliferation T1	> 1	1.70	1.13	3.63	Compliant
	Proliferation T2	> 1	1.36	0.69	3.27	Not compliant
	Proliferation T4	> 1	1.22	0.22	3.12	Not compliant
	Potency T0 (%)	> 25%	67	63	68	Compliant
	Potency T1 (%)	> 25%	73	57	54	Compliant
	Potency T2 (%)	> 25%	66	Not done	55	NA
	Potency T4 (%)	> 25%	66	Not done	60	NA
	Morphology T0	Fibroblastic	OK	OK	OK	Compliant
	Morphology T1	Fibroblastic	OK	OK	OK	Compliant
	Morphology T2	Fibroblastic	OK	OK	OK	Compliant
	Morphology T4	Fibroblastic	OK	OK	OK	Compliant

Table S4. Short-term stability of fresh MSC products after formulation and 1, 2, 3 and 4 hours thereafter. Results for short-term stability of 3 different populations of fresh MSC are shown after formulation and 1, 2 and until 4 hours thereafter. Recovery represents the ratio of thawed MSC/frozen MSC at the different times. Proliferation is defined as the ratio of number of cells after one week of post-thawing culture / number of cells recovered immediately after thawing. Potency is calculated as inhibition of activated PBMC proliferation in co-culture with MSC.

**Validation run number**			**1**	**2**	**3**	
	**Tests**	**Specification**	**Results**	**Results**	**Results**	**Conformity**
**Harvest**	Identity (%)	CD90 > 95%	99.5	99.0	99.7	Compliant
		CD105 > 95%	98.7	99.3	99.5	Compliant
		CD73 > 95%	99.0	99.5	99.5	Compliant
	Purity (%)	CD14 < 2%	0	0	0	Compliant
		CD34 < 2%	0.30	0	0	Compliant
		CD45 < 2%	0	0	0	Compliant
		CD3 < 1%	0	0	0	Compliant
		Total < 2%	0.30	0	0	Compliant
	Morphology	Fibroblastic	Fibroblastic	Fibroblastic	Fibroblastic	Compliant
**T0**	Numeration (10^6/mL)	NA	3.48	4.28	4.00	Compliant
	Viability (%)	≥ 80%	94	96	94	Compliant
	Potency (%)	> 25%	49	49	47	Compliant
	Proliferation (7 days)	> 1	2.6	2.5	1.9	Compliant
**T1**	Recovery (%/T0)	≥ 80%	111	100	105	Compliant
	Viability (%)	≥ 80%	94	97	95	Compliant
	Potency (%)	> 25%	42	58	39	Compliant
	Proliferation (7 days)	> 1	3.1	2.1	1.9	Compliant
**T2**	Recovery (%/T0)	≥ 80%	104	106	114	Compliant
	Viability (%)	≥ 80%	93	96	95	Compliant
	Potency (%)	> 25%	41	38	25	Compliant
	Proliferation (7 days)	> 1	2.9	1.8	1.6	Compliant
**T3**	Recovery (%/T0)	≥ 80%	118	100	99	Compliant
	Viability (%)	≥ 80%	93	94	87	Compliant
	Potency (%)	> 25%	34	33	34	Compliant
	Proliferation (7 days)	> 1	2.8	1.7	1.3	Compliant
**T4**	Recovery (%/T0)	≥ 80%	101	94	113	Compliant
	Viability (%)	≥ 80%	94	95	96	Compliant
	Potency (%)	> 25%	48	42	28	Compliant
	Proliferation (7 days)	> 1	2.7	1.5	2.6	Compliant
